# Supplementary material for: Breastfeeding Practices among Adolescent Mothers and Associated Factors in Bangladesh (2004–2014)
Source: Nutrients. 2021 Feb 8;13(2):557. doi: 10.3390/nu13020557 (PMC7915163; doi:10.3390/nu13020557)
Supplement: Supplementary file 1 [file nutrients-13-00557-s001.pdf]

**Supplementary Table 1**. Definition and categorisation of potential variables used in the study

| Independent variables | Categorisation |
|---|---|
| ***Individual-level factors*** | |
| Maternal religion | In the 2 following categories: (1=Islam; 2=others) |
| Maternal working status | In the 2 following categories: (1=not working; 2=working) |
| Maternal education | In the 3 following categories: (1=No education; 2=Primary; 3=Secondary or higher) |
| Paternal education | In the 3 following categories: (1=No education; 2=Primary; 3=Secondary or higher) |
| Husband's occupation | In the 3 following categories: (1=Non-agricultural; 2=Agricultural; 3=Not working) |
| Adolescent mother's age | In the 2 following categories: (1= 12-18 years; 2=18-19 years) |
| Marital status | In the 2 following categories: (1= Currently married; 2=divorced/separated/widow) |
| Birth order | In the 2 following categories: (1= First-born; 2=2nd-4th born) |
| Preceding birth interval | In the 2 following categories: (1= No previous birth; 2=Yes) |
| Sex of baby | In the 2 following categories: (1=Male; 2=Female) |
| Number of living children | In the 2 following categories: (1= 1 child; 2=2-4 children) |
| Age of child (months) | In the 4 following categories: (1=0-5 months; 2=6-11 months; 3=12-17 months; 4=18-23 months) |
| | |
| Combined mode and place of delivery | In the 3 following categories: (1=Caesarean; 2=Vaginal & Health Facility; 3=Home) |
| Type of delivery assistance | In the 2 following categories: (1=Health professional[&]; 2=Non-health professional) |
| Antenatal Clinic visits | In the 4 following categories: (1) ≥8 antenatal care visits, (2) 4-7 antenatal care visits, and (3) 1-3 antenatal care visits (4) no antenatal care visits from a skilled provider for the most recent birth |
| Postnatal check-up | In the 3 following categories: (1=0-2 days; 2=After 2 days; 3=No postnatal check-up) |
| Adolescent mother BMI | In the 3 following categories: (1= <18.5; 2= 18.5-24.9; 3= ≥25) |
| ***Exposure to Media*** | |
| Adolescent mother reading newspapers | In the 2 following categories: (1= Not at all; 2=Yes) |
| Adolescent mother listening to radio | In the 2 following categories: (1= Not at all; 2=Yes) |
| Adolescent mother watching television | In the 2 following categories: (1= Not at all; 2=Yes) |
| ***Household-level factors*** | |
| Household wealth Index | hv217 (the household wealth index factor score) constructed by DHS based on a selected set of household assets. In quintiles, 1= richest; 2=richer; 3= middle; 2=poorer and 1= poorest) |
| Decision-making (Autonomy) | In the 3 following categories: (1= No Decision (0 score); 2= Some Decisions (1-2 scores); and 3= All Decisions (3 scores)) |
| ***Community-level factors*** | |
| Residence | In the 2 following categories:(1=urban; 2=rural) |

| | |
|---|---|
| Geographical Region without Rangpur | In the 6 following categories: (1=Barisal; 2=Chittagong; 3=Dhaka; 4=Khulna; 5=Rajshahi; 6=Sylhet) |
| Geographical Region with Rangpur | In the 6 following categories: (1=Barisal; 2=Chittagong; 3=Dhaka; 4=Khulna; 5=Rajshahi; 6=Sylhet; 7=Rangpur) |

&= doctor; nurse/midwife/paramedics, family welfare visitor medical assistant/community medical officer/ health assistant.

**Supplementary Table 2. key Breastfeeding indicators by year among children 0-23 months of age, Bangladesh 2004-2014 (n=2554)**

| **Year of survey** | **2004 (N*=614)** | | | **2007 (N*=521)** | | | **2011 (N*=693)** | | | **2014 (N*=726)** | | |
|---|---|---|---|---|---|---|---|---|---|---|---|---|
| **Indicator** | **N*** | **n*** | **Rate (95% CI)** | **N*** | **n*** | **Rate (95% CI)** | **N*** | **n*** | **Rate (95% CI)** | **N*** | **n*** | **Rate (95% CI)** |
| **Early initiation of breastfeeding rate[a]** | | | | | | | | | | | | |
| Yes | 614 | 153 | 24.9 (21.3, 28.8) | 521 | 226 | 43.3 (38.5, 48.3) | 693 | 326 | 47.1 (43.0, 51.2) | 726 | 374 | 51.4 (46.2, 56.7) |
| **Bottle-feeding rate[a]** | | | | | | | | | | | | |
| Yes ^ | 614 | 93 | 15.2 (12.4, 18.6) | * | * | * | 693 | 99 | 14.2 (11.5, 17.4) | 726 | 127 | 17.5 (13.3, 22.6) |
| **Exclusive breastfeeding rate[c]** | | | | | | | | | | | | |
| Yes | 187 | 83 | 44.1 (36.5, 52.1) | 132 | 59 | 44.9 (35.5, 54.8) | 214 | 144 | 67.1 (59.3, 74.1) | 212 | 109 | 51.4 (41.0, 61.7) |
| **Predominant breastfeeding rate[c]** | | | | | | | | | | | | |
| Yes | 187 | 45 | 24.3 (18.2, 31.5) | 132 | 22 | 17.0 (11.0, 25.4) | 214 | 25 | 11.7 (7.6, 17.5) | 212 | 36 | 17.1 (11.5, 24.7) |

**Supplementary Table 3 Individual, household and community and level characteristics of children 0-23 months of age by year of survey, Bangladesh 2004-2014.**

| Characteristic | **2004** (n=614) | | **2007** (n=521) | | **2011** (n=693) | | **2014** (n=726) | |
|---|---|---|---|---|---|---|---|---|
| | **n** | % | **n** | % | **n** | % | **n** | % |
| *Individual level factors* | | | | | | | | |
| **Mother's religion** | | | | | | | | |
| Islam | 572 | 93.2 | 491 | 94.2 | 636 | 91.9 | 675 | 92.9 |
| Others$ | 41 | 6.6 | 31 | 5.9 | 56 | 8.1 | 51 | 7.1 |
| **Mother's working status** | | | | | | | | |
| Non-working | 564 | 91.9 | 443 | 85.0 | 665 | 96.0 | 647 | 89.2 |
| Working (past 12 months) | 49 | 8.1 | 78 | 15.0 | 27 | 4.0 | 79 | 10.8 |
| **Mother's education** | | | | | | | | |
| No education | 103 | 16.7 | 12 | 11.5 | 68 | 9.8 | 37 | 5.1 |
| Primary | 228 | 37.2 | 31 | 30.9 | 203 | 29.3 | 219 | 30.1 |
| Secondary and higher | 282 | 46.0 | 58 | 57.6 | 422 | 60.9 | 470 | 64.8 |
| **Partner's education** | | | | | | | | |
| No education | 202 | 32.9 | 146 | 28.0 | 132 | 19.0 | 116 | 16.0 |
| Primary | 366 | 59.7 | 169 | 32.4 | 248 | 35.8 | 281 | 38.7 |
| Secondary and higher | 46 | 7.4 | 205 | 39.2 | 313 | 45.2 | 329 | 45.3 |
| **Partner's occupation** | | | | | | | | |
| Non-agricultural | 396 | 64.5 | 367 | 70.5 | 412 | 59.5 | 407 | 56.1 |
| Agricultural | 196 | 32.0 | 141 | 27.1 | 168 | 24.3 | 161 | 22.2 |
| Not working | 22 | 3.5 | 13 | 2.5 | 113 | 16.3 | 158 | 21.8 |
| **Mother's age** | | | | | | | | |
| 12- 18 years | 304 | 49.5 | 198 | 38.0 | 270 | 38.9 | 290 | 39.9 |
| 18-19 years | 310 | 50.5 | 323 | 62.0 | 423 | 61.1 | 436 | 60.1 |
| **Mother's marital status** | | | | | | | | |
| Currently married | 606 | 98.7 | 511 | 98.0 | 690 | 99.7 | 720 | 99.1 |
| Formerly married^ | 8 | 1.3 | 10 | 2.0 | 2 | 0.3 | 6 | 0.9 |
| **Birth order** | | | | | | | | |
| First-born | 452 | 73.7 | 414 | 79.3 | 568 | 82.0 | 649 | 89.4 |
| $2^{nd}$-$4^{th}$ | 162 | 26.3 | 108 | 20.7 | 125 | 18.0 | 77 | 10.6 |
| **Preceding birth interval** | | | | | | | | |
| No previous birth | 453 | 73.8 | 414 | 79.3 | 572 | 82.5 | 649 | 89.4 |
| Yes | 161 | 26.2 | 108 | 20.7 | 121 | 17.5 | 77 | 10.6 |
| **Sex of baby** | | | | | | | | |
| Male | 301 | 49.1 | 257 | 49.2 | 352 | 50.9 | 393 | 54.1 |
| Female | 312 | 50.9 | 265 | 50.8 | 340 | 49.1 | 333 | 45.9 |
| **Number of living children** | | | | | | | | |
| 1 | 476 | 77.7 | 435 | 83.4 | 587 | 84.8 | 659 | 90.7 |
| 2-4 | 137 | 22.3 | 87 | 16.7 | 105 | 15.2 | 67 | 9.3 |
| **Age of child (in months)** | | | | | | | | |
| 0-5 | 187 | 30.5 | 132 | 25.3 | 214 | 30.9 | 212 | 29.2 |

| | | | | | | | | |
|---|---|---|---|---|---|---|---|---|
| 6-11 | 161 | 26.2 | 143 | 27.3 | 201 | 29.1 | 212 | 29.2 |
| 12-17 | 161 | 26.2 | 113 | 21.7 | 175 | 25.3 | 179 | 24.7 |
| 18-23 | 105 | 17.1 | 134 | 25.7 | 102 | 14.8 | 123 | 16.9 |
| **Combined mode and place of delivery (n= 2548)** | | | | | | | | |
| Caesarean & Health Facility | 545 | 88.9 | 438 | 83.9 | 485 | 70.0 | 466 | 64.2 |
| Vaginal & Health Facility | 40 | 6.4 | 49 | 9.4 | 112 | 16.2 | 106 | 14.5 |
| Home | 23 | 3.7 | 35 | 6.7 | 96 | 13.9 | 155 | 21.3 |
| **Type of delivery assistance (n= 2543)** | | | | | | | | |
| Health professional | 57 | 9.4 | 81 | 15.6 | 175 | 25.3 | 227 | 31.3 |
| Non-health professional | 555 | 90.4 | 432 | 82.8 | 516 | 74.5 | 499 | 68.7 |
| **Antenatal Clinic visits** | | | | | | | | |
| 8+ | 18 | 3.0 | 12 | 2.3 | 32 | 4.6 | 42 | 5.8 |
| 4-7 | 81 | 13.2 | 90 | 17.3 | 161 | 23.3 | 176 | 24.2 |
| 1-3 | 267 | 43.6 | 234 | 44.9 | 299 | 43.1 | 379 | 52.1 |
| None | 247 | 40.3 | 185 | 35.5 | 201 | 29.0 | 130 | 17.9 |
| **Postnatal check-up** | | | | | | | | |
| 0-2 days | 74 | 12.1 | 115 | 22.1 | 144 | 20.8 | 297 | 40.9 |
| After 2 days | 38 | 6.3 | 56 | 10.7 | 67 | 9.7 | 134 | 18.4 |
| No postnatal check-up | 501 | 81.6 | 350 | 67.2 | 482 | 69.6 | 295 | 40.7 |
| **Mother's BMI** | | | | | | | | |
| <18.5 | 217 | 35.4 | 143 | 27.4 | 199 | 28.7 | 195 | 26.9 |
| 18.5-24.9 | 377 | 61.4 | 360 | 69.1 | 458 | 66.1 | 480 | 66.1 |
| 25+ | 4 | 0.7 | 11 | 2.2 | 28 | 4.0 | 48 | 6.6 |
| **Exposure to Media** | | | | | | | | |
| **Mothers reading Newspapers** | | | | | | | | |
| Not at all | 507 | 82.7 | 458 | 87.9 | 603 | 87.1 | 627 | 86.4 |
| Yes[#] | 106 | 17.3 | 63 | 12.1 | 89 | 12.9 | 97 | 13.3 |
| **Mothers listening to radio** | | | | | | | | |
| Not at all | 304 | 49.5 | 359 | 68.8 | 612 | 88.3 | 675 | 93.0 |
| Yes[#] | 310 | 50.5 | 163 | 31.2 | 81 | 11.7 | 51 | 7.0 |
| **Mothers watching TV** | | | | | | | | |
| Not at all | 257 | 41.8 | 246 | 47.1 | 245 | 35.4 | 308 | 42.5 |
| Yes[#] | 356 | 57.9 | 276 | 52.9 | 447 | 64.6 | 418 | 57.5 |
| ***Household level factors*** | | | | | | | | |
| **Household wealth Index** | | | | | | | | |
| Richest | 93 | 15.1 | 74 | 14.1 | 160 | 23.0 | 171 | 23.5 |
| Richer | 164 | 26.7 | 93 | 17.8 | 142 | 20.5 | 143 | 19.7 |
| Middle | 143 | 23.3 | 123 | 23.7 | 114 | 16.5 | 126 | 17.4 |
| Poorer | 95 | 15.5 | 167 | 32.1 | 137 | 19.8 | 103 | 14.2 |
| Poorest | 119 | 19.3 | 64 | 12.3 | 139 | 20.1 | 183 | 25.3 |
| **Decision-making (Autonomy)** | | | | | | | | |
| No Decision (0 score) | 329 | 53.7 | 238 | 45.6 | 248 | 35.8 | 246 | 33.9 |
| Some Decisions (1-2 scores) | 188 | 30.7 | 177 | 34.0 | 213 | 30.7 | 223 | 30.7 |
| All Decisions (2 scores) | 96 | 15.6 | 106 | 20.4 | 232 | 33.5 | 257 | 35.4 |

| | | | | | | | | |
|---|---|---|---|---|---|---|---|---|
| *Community level factors* | | | | | | | | |
| **Residence** | 110 | 18.0 | 87 | 16.7 | 141 | 20.3 | 177 | 24.5 |
| Urban | 503 | 82.0 | 434 | 83.3 | 552 | 79.7 | 549 | 75.6 |
| Rural | | | | | | | | |
| **Geographical Region without Rangpur** | | | | | | | | |
| Barisal | 37 | 5.987 | 28 | 5.443 | 39 | 6.5 | 41 | 6.3 |
| Chittagong | 130 | 21.16 | 98 | 18.84 | 145 | 24.5 | 161 | 24.9 |
| Dhaka | 162 | 26.42 | 167 | 32.07 | 202 | 34.0 | 260 | 40.2 |
| Khulna | 71 | 11.52 | 43 | 8.258 | 90 | 15.2 | 62 | 9.6 |
| Rajshahi | 186 | 30.28 | 150 | 28.72 | 91 | 15.4 | 68 | 10.4 |
| Sylhet | 28 | 4.63 | 35 | 6.667 | 27 | 4.5 | 56 | 8.6 |
| **Geographical Region with Rangpur** | | | | | | | | |
| Barisal | | | | | 39 | 5.6 | 41 | 5.6 |
| Chittagong | | | | | 145 | 21.0 | 161 | 22.1 |
| Dhaka | | | | | 202 | 29.1 | 260 | 35.9 |
| Khulna | | | | | 90 | 13.0 | 62 | 8.5 |
| Rajshahi | | | | | 91 | 13.2 | 68 | 9.3 |
| Sylhet | | | | | 27 | 3.9 | 56 | 7.6 |
| Rangpur | | | | | 99 | 14.3 | 79 | 10.9 |

Supplementary Table 4. Distribution of breastfeeding by child age in months (2004-2014)

| Age in months | Exclusively BF | BF + water | BF+ Liquids/Juice | BF+ Other milk | BF+ Complimentary feeds | No BF | Total |
|---|---|---|---|---|---|---|---|
| 0 | 86.8 | 2.9 | 3.1 | 7.2 | 0.0 | 0.0 | 100 |
| 1 | 71.8 | 12.4 | 2.7 | 7.3 | 4.8 | 1.1 | 100 |
| 2 | 56.6 | 15.6 | 3.7 | 16.2 | 5.6 | 2.3 | 100 |
| 3 | 53.5 | 9.4 | 8.5 | 21.3 | 7.2 | 0.0 | 100 |
| 4 | 43.3 | 16.4 | 5.8 | 19.4 | 14.0 | 1.2 | 100 |
| 5 | 19.0 | 15.1 | 4.9 | 21.9 | 39.0 | 0.2 | 100 |
| 6 | 11.4 | 18.6 | 5.0 | 11.2 | 49.8 | 4.0 | 100 |
| 7 | 3.0 | 14.5 | 3.0 | 8.7 | 69.8 | 1.1 | 100 |
| 8 | 2.0 | 5.5 | 2.5 | 9.7 | 78.3 | 2.0 | 100 |
| 9 | 0.0 | 3.9 | 1.8 | 0.8 | 89.4 | 4.1 | 100 |
| 10 | 0.0 | 5.5 | 2.2 | 2.7 | 84.5 | 5.2 | 100 |
| 11 | 0.8 | 2.8 | 1.2 | 0.4 | 93.6 | 1.1 | 100 |
| 12 | 0.4 | 2.6 | 3.1 | 2.6 | 88.2 | 3.1 | 100 |
| 13 | 1.4 | 4.3 | 0.0 | 0.0 | 88.3 | 6.0 | 100 |
| 14 | 1.0 | 0.7 | 0.0 | 2.6 | 94.6 | 1.1 | 100 |
| 15 | 0.3 | 1.4 | 0.0 | 8.1 | 84.8 | 5.5 | 100 |
| 16 | 0.0 | 0.8 | 0.0 | 1.6 | 91.3 | 6.3 | 100 |
| 17 | 0.0 | 2.8 | 0.0 | 0.0 | 96.0 | 1.2 | 100 |
| 18 | 0.0 | 1.5 | 0.0 | 0.0 | 96.6 | 1.8 | 100 |
| 19 | 0.0 | 1.0 | 0.0 | 0.0 | 90.5 | 8.5 | 100 |
| 20 | 0.0 | 0.2 | 0.0 | 0.0 | 93.7 | 6.1 | 100 |
| 21 | 1.6 | 0.9 | 0.0 | 0.9 | 89.5 | 7.2 | 100 |
| 22 | 0.0 | 0.0 | 0.0 | 2.0 | 85.4 | 12.6 | 100 |
| 23 | 0.0 | 3.4 | 0.0 | 0.0 | 87.5 | 9.2 | 100 |
